# Carbon Nanotube Hydrogel Electrodes for High-Fidelity Intra-Aural EEG in Wearable Neurotechnology

**DOI:** 10.3390/s26102973

**Published:** 2026-05-08

**Authors:** Alexandra-Ștefania Mihai, Ana-Maria Iordache, Liliana Vereștiuc, Isabella Nacu, Oana Geman

**Affiliations:** 1 Department of Computers, Electronics, and Automation, Faculty of Electrical Engineering and Computer Science, “Ștefan cel Mare” University of Suceava, 720229 Suceava, Romania; 2 National Institute for Research and Development in Optoelectronics—INOE 2000, 409 Atomistilor St., 077125 Măgurele, Romania; ana.iordache@inoe.ro; 3 Department of Biomedical Sciences, Faculty of Medical Bioengineering, Grigore T. Popa University of Medicine and Pharmacy, 9-13 M. Kogalniceanu St., 700454 Iași, Romania; liliana.verestiuc@umfiasi.ro (L.V.);; 4 Data Science and AI Group, Department of Computer Engineering, Chalmers University of Technology, 412 96 Gothenburg, Sweden; 5 Data Science and AI Group, Department of Computer Engineering, University of Gothenburg, 405 30 Gothenburg, Sweden

**Keywords:** intra-auricular EEG, electrochemical impedance spectroscopy, electrodes for intra-auricular EEG

## Abstract

Electrical monitoring of brain activity can be performed discreetly and continuously over long periods of time using intra-auricular electroencephalography (intra-auricular EEG), a promising technique suitable for subjects who are difficult to monitor, such as newborns or patients with neurological conditions requiring discreet but long-term neurophysiological assessment. The concept of intra-aural EEG can be realized through the development of systems that include wearable sensors, whose performance critically depends on the development of biocompatible electrode materials that exhibit low impedance and can maintain and provide stable contact between the electrode and the epithelial tissue. Based on our previous work on carbon nanotube (CNT)-based hydrogel composites for intra-aural EEG electrodes, this study focuses on the electrochemical characterization of hydrogels initially prepared from gelatin methacrylate (GelMA)/2-hydroxyethyl methacrylate (HEMA) doped with varying concentrations of CNTs (0–3 wt%). In the present study, the materials obtained in the first stage were evaluated using electrochemical impedance spectroscopy (EIS) under both liquid and dry conditions, supplemented by measurements of hydration capacity. The results show that the composite with 3% CNT content exhibits suitable properties, making the material making the 3 wt% CNT formulation a promising platform for the further development of 3D-printable hydrogel electrodes for intra-aural EEG applications. Equivalent circuit modeling reveals improved ionic and electronic conductivity compared to the undoped hydrogel, attributed to better CNT dispersion and polymer crosslinking. This work provides insights into the structure–property relationships of CNT–hydrogel composites and lays the foundation for the further development of a 3D-printed and in vitro/in vivo validated prototype of intra-aural EEG sensors.

## 1. Introduction

The standard in neuromonitoring of the brain’s bioelectric activity is electroencephalography (EEG), a technique based on recording potential differences using electrodes placed on the scalp [1]. However, with innovative approaches in the field of neurotechnology, wearable electroencephalography technologies have also made significant progress, leading to the development of the concept of intra-auricular EEG. Through this aforementioned technique, signals transmitted by the brain are captured and recorded using electrodes located in the ear canal or in the periauricular region. This new approach facilitates the discreet, non-invasive, and continuous monitoring of brain activity [2].

The study [2] lays the groundwork for the concept of intra-auricular EEG, as the authors proposed, implemented, and tested the first platform for recording EEG directly from inside the ear canal. At the same time, the authors succeeded in demonstrating the feasibility of the concept by simultaneously comparing signals captured from the ear with the classical EEG recording method, which involves the use of electrodes placed on the scalp, using the classical alpha-attenuation paradigm (eyes closed/open). The prototype used consisted of a custom earplug featuring two built-in Ag/AgCl electrodes that were connected to the same amplifier as the scalp electrodes. The results obtained consisted of EEG signals comparable to those recorded conventionally: the signals recorded at the ear had a signal-to-noise ratio (SNR) similar to that of the signal captured by the electrodes placed on the scalp, although with a lower amplitude and slightly higher noise. Additionally, good coherence with nearby scalp electrodes (T7 and M1) and reduced magnetic artifacts were observed. Thus, this study demonstrated the viability of the intra-aural EEG concept for continuous and discrete recording.

Traditional pediatric neurology investigations rely on brain ultrasound and neurological examination of gross motor skills [3,4,5,6]. These methods are considered the gold standard for neonatal assessment in Romania. However, performing brain ultrasound on restless infants is difficult, and the examination of general movements is subjective, depending on the physician’s experience. Furthermore, these methods provide only point-in-time measurements and cannot be used to assess the progression of neurological diseases [7]. Wearable technology offers the possibility of continuously monitoring a person’s physiological brain activity and identifying autonomic patterns associated with psychiatric conditions, such as autism spectrum disorder (ASD), anxiety, and mood disorders [8,9], acting as an early warning system. In newborns, the in-ear EEG approach is being investigated specifically for the detection of subclinical seizures and the assessment of brain maturity, as it is compatible with the comfort and safety requirements of neonatology.

Despite its many advantages, the intra-auricular EEG also faces a number of inherent challenges stemming from the anatomical variability of the external auditory canal. Unlike conventional EEG, where electrodes are placed on a relatively flat and easily accessible surface, intra-aural electrodes must capture signals from an area with much lower spatial resolution, as the intra-aural microenvironment is narrow, curved, and in constant motion. The limited and fluctuating humidity of the ear canal can often lead to partial dehydration of the electrode-skin interface during prolonged wear, reducing the long-term stability of the recorded signals. A decisive factor in signal acquisition using in-ear electrodes is the high variability of the electrode-skin impedance caused by the presence and accumulation of earwax. Earwax acts as an insulating layer that, with prolonged wear, can increase impedance by several orders of magnitude, directly degrading the quality of the recorded signal and the SNR [10]. Jaw movement, head rotation, or even subtle facial expressions result in motion artifacts that cause micro-displacements of the electrode, thereby introducing low-frequency noise that overlaps with important brain rhythms [11,12,13,14].

The implementation of systems for intra-aural EEG recordings begins with the integration of advanced biomaterials into the final device architecture. To achieve morphological optimization and electronic miniaturization of the final devices, it is necessary to implement and utilize a multidimensional approach that includes the development of advanced materials with characteristics aimed at biocompatibility and moisture retention. In this regard, carbon nanotube-doped hydrogels have been proposed in this paper—a feasible strategy that reduces the limitations of intra-auricular EEG and offers mechanical flexibility, increased skin compatibility, and low, stable interfacial impedance [15]. As an innovative concept in neuroscience, intra-auricular EEG opens new perspectives on the biomaterials used, representing a new intersection between advanced materials science and applied neuroengineering. The process of developing a wearable EEG sensor begins with materials science, and selecting the appropriate material for the sensor is of major importance. This study aims to introduce a new material: an electrode made of hydrogel doped with carbon nanotubes to meet both conductivity requirements and the constraints imposed by the manufacturing process, particularly those associated with 3D printing technologies [15,16,17,18].

Conductive hydrogels offer a number of advantages, including low interfacial impedance and the ability to be used for extended periods of time. A concrete example of this is [19]. In this study, the authors developed a hydrogel suitable for applications involving the use of intra-auricular EEG electrodes. The synthesized hydrogel exhibits high conductivity and an impedance of up to 8 Ω. Furthermore, using these ionic hydrogel-based electrodes, recordings were made over a minimum period of 8 h. The acquired signal demonstrated long-term stability, and the electrodes did not cause any damage to the epithelial tissue.

Another class of hydrogels that demonstrated high long-term stability (>8 h) consists of injectable and self-adhesive hydrogels [20]. Unlike classic dry electrode variants made of Ag/AgCl, alternative approaches for in-ear electrodes are based on dry electrodes containing CNT/PDMS (polydimethylsiloxane) composites, initially introduced in [21] and subsequently improved in [22], in optimized variants with integrated bioamplifiers, which are softer and adaptable to the ear canal space. Compared to hydrogel-based systems, CNT/PDMS composites feature a discreet design and acceptable mechanical flexibility, but exhibit high sensitivity to motion artifacts and increased baseline impedance.

Although these approaches have successfully demonstrated low impedance and operational stability exceeding 8 h under controlled conditions, most existing hydrogel-based auricular EEG solutions rely on ionic conductors or ex situ fabricated composites that still require external gels or lack comprehensive electrochemical characterization under both hydrated and dry conditions. Furthermore, few studies integrate comprehensive biological, mechanical, and interfacial electrochemical data with a clear path toward customized, 3D-printable intra-aural devices.

This study focuses on analyzing the effect of doping a GelMA/HEMA hydrogel matrix with single-walled carbon nanotubes (SWCNTs) (0–3 wt%) on its electrochemical impedance and hydration stability. Recent advances in 3D-printed conductive hydrogels and bio-electrodes [23,24,25] have highlighted the potential of such materials for customized, wearable neurotechnology devices. Following systematic characterization of hydration capacity and detailed electrochemical impedance spectroscopy (EIS) testing under both liquid (PBS) and dry conditions, the formulation containing 3 wt% CNTs emerges as a promising composition, exhibiting the lowest charge-transfer resistance and the highest hydration capacity among the tested samples.

While the present work focuses on electrochemical and hydration characterization at the material level, future studies will include prototype fabrication, 3D printing of customized in-ear electrodes, and in situ validation with real EEG signals. Furthermore, although PBS was used as the control electrolyte in this study, future experiments will incorporate more realistic testing conditions (e.g., synthetic sebum and cerumen-coated interfaces) to better reflect the challenges of the ear canal microenvironment.

## 2. Preliminary Results

In our initial study [26], we synthesized conductive hydrogel composites suitable for intra-aural EEG electrodes. The materials were prepared from 10% gelatin methacrylate (GelMA) and 2-hydroxyethyl methacrylate (HEMA). Single-walled carbon nanotubes (SWCNTs) purchased from Sigma-Aldrich (product no. 805033-25G) were used. According to the manufacturer, the SWCNTs have the following characteristics: carbon content ≥85% (>70% as SWNT), diameter 1.3–2.3 nm, tubular morphology.

The SWCNTs were dispersed in the GelMA/HEMA precursor solution by ultrasonication. The composites were doped with 0 wt% (Control), 1 wt% (Sample 1), 2 wt% (Sample 2), and 3 wt% (Sample 3) SWCNTs (Table 1). Cross-linking was performed via radical polymerization using an APS/TEMED redox system under ambient conditions. The detailed synthesis protocol, including exact quantities, sonication parameters, and purification steps, is provided in [26].

The obtained samples presented smooth surfaces suitable for skin contact. With increasing SWCNT content, a progressive darkening and textural change were observed. Due to the nanoscale dimensions of individual SWCNTs (1.3–2.3 nm), optical microscopy (if performed) cannot resolve single nanotubes and mainly reveals larger agglomerates.

Nevertheless, the significant improvement in electrochemical performance and the higher hydration capacity at 3 wt% SWCNT provide strong indirect evidence of a more effective conductive network at this loading. A detailed morphological characterization (e.g., SEM or TEM) is required for quantitative assessment of dispersion uniformity and will be conducted in future studies.

Cytocompatibility tests were conducted via direct contact with human dermal fibroblasts. The results demonstrated cell viability above the ISO 10993 [27] thresholds after 24 h.

**Figure 1:**
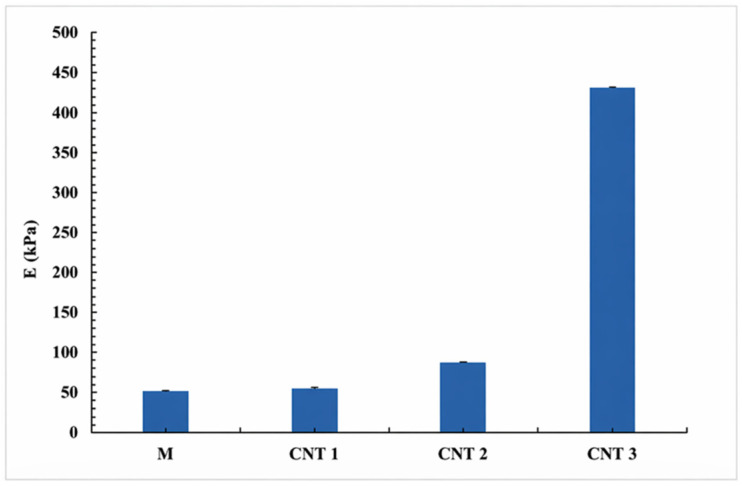
Young’s modulus of GelMA/HEMA hydrogels as a function of CNT concentration (data from [26]). Values are presented as mean ± standard deviation (*n* = 3).

The mechanical characterization, previously reported in [26], showed a progressive increase in stiffness with SWCNT loading. As shown in Figure 1, Young’s modulus increased from approximately 50 kPa for the undoped hydrogel (M) to ~55 kPa (CNT 1%), ~85 kPa (CNT 2%), and ~430 kPa for the 3 wt% SWCNT sample—representing an approximately 8.6-fold enhancement compared to the control.

This significant reinforcement at relatively high CNT content, although contrasting with the more typical peak at lower loadings (<1–2 wt%) reported in many CNT–polymer composites [28,29], can be attributed to the effective dispersion method employed (ultrasonic dispersion in the GelMA/HEMA precursor solution before redox cross-linking) and favorable interfacial interactions within this specific hydrophilic matrix. It should be noted that such behavior is system-specific and may not be generalizable to other CNT–polymer systems.

Collectively, the biological and mechanical data from the previous work established that the GelMA/HEMA/CNT composites possess suitable cytocompatibility and tunable mechanical properties for intra-aural EEG electrodes. Building upon these promising results, the present study focuses on the electrochemical characterization (hydration capacity and electrochemical impedance spectroscopy in both liquid and dry conditions) of the same hydrogel formulations to identify the optimal composition for low-impedance, stable electrode–electrolyte interfaces.

## 3. Materials and Methods

Electrochemical impedance spectroscopy (EIS) measurements were performed using an OrigaLys electrochemical workstation (OrigaLys, Rillieux-la-Pape, France). Measurements were performed in the following range: 100 kHz–10 mHz, AC current range 500 µA, AC current amplitude 5 mV, frequency per decade 10 Hz. The hydrogel was conditioned in a phosphate-buffered saline solution (PBS) for two hours. The hydrogel samples were conditioned in phosphate-buffered saline (PBS, pH 7.4) for two hours prior to EIS measurements to achieve initial hydration equilibrium. PBS was chosen because it provides several advantages: it is isotonic and appropriate for biocompatibility experiments, it maintains the swelling properties of the hydrogel, and it promotes cross-linking.

Two types of EIS measurements were performed:

(i) In liquid, using a three-electrode assembly consisting of a reference electrode (Ag/AgCl), a counter electrode (Pt wire), and the working electrode (hydrogel connected via a cable with alligator clips); the supporting electrolyte is a phosphate-buffered saline solution, pH = 7.4, from Alfa Aesar (Thermo Scientific Chemicals, Waltham, MA, USA).
(ii) Under dry conditions, using an adjustable 2-electrode platform designed for EIS measurements (a hollow round perfluoroalkoxy chamber equipped with 2 round steel electrodes connected to the Origalys potentiostat via simple connecting cables; the counter electrode and the reference electrode are electrically coupled; the hydrogel sample is placed between the two electrodes). The dimensions of the cell are diameter = 5 cm, depth = 2 cm. The pistons are both moving independently and can be controlled separately. Samples (≈1 cm diameter and ≈4 mm thickness) were loaded in the EIS cell and placed between the electrodes. In order to control the compression force on the hydrogels, we used the electrodes of the cell as an anvil. The force applied to the sample was controlled by tightening the screws connected to the electrodes.

**Figure 2:**
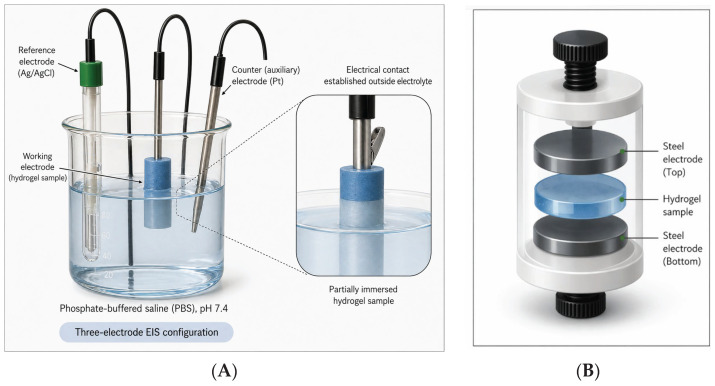
Electrochemical setup for EIS measurements: (**A**) in liquid conditions and (**B**) in dry conditions.

The setups for the two measurements are shown in Figure 2.

EIS measurements in liquid conditions were performed in PBS (pH 7.4) as a standardized physiological electrolyte to ensure reproducibility and comparability between samples. However, it should be noted that this simplified medium does not replicate the complex composition of the ear canal, particularly the insulating properties of human cerumen (earwax) and the lipid-rich sebum environment, which can significantly increase interfacial impedance in real-world conditions.

## 4. Results

### 4.1. Hydration Capacity of Hydrogels

The degree of hydration was determined using the equation:

$$Degree\;of\;hydration\;\left(\%\right)\frac{{mass}_{final}-{mass}_{initial}}{{mass}_{final}}\;*\;100$$

The results are presented in Table 2.

The degree of hydration of the hydrogels varies non-monotonically with the amount of incorporated SWCNTs. The hydration capacity decreases to 5.4%/cm^2^ for Sample 2 (2 wt% SWCNT), followed by a sharp increase to 7.4%/cm^2^ for Sample 3 (3 wt% SWCNT), slightly exceeding the value of the undoped reference hydrogel (7.2%/cm^2^).

This non-monotonic behavior can be explained by the competing effects of CNT dispersion/agglomeration state and its influence on the polymer network microstructure. At intermediate concentration (2 wt%), SWCNTs tend to form larger agglomerates driven by strong van der Waals interactions and π–π stacking. These agglomerates act as physical obstacles within the GelMA/HEMA matrix, increasing the local effective cross-linking density and creating hydrophobic microdomains that restrict polymer chain mobility and reduce the free volume available for water molecules, thereby inhibiting swelling. In contrast, at 3 wt% SWCNT, the higher nanofiller loading appears to promote the formation of a more interconnected porous structure. The presence of CNT aggregates disrupts the polymer network in a way that increases overall porosity and creates additional pathways for water penetration, leading to enhanced water retention compared to the 2 wt% sample. This interpretation aligns with several literature reports on CNT–hydrogel composites, where non-monotonic swelling behavior is observed and often attributed to changes in microstructure and pore formation induced by nanofiller agglomeration at higher concentrations [15,16,17,30,31].

It should be noted that pristine SWCNTs possess a hydrophobic surface. No oxidative functionalization (e.g., introduction of carboxyl groups) was performed in this study. Therefore, the improved hydration at 3 wt% is unlikely to result from increased hydrophilicity of the nanotubes themselves, but rather from morphological changes in the composite (increased porosity due to aggregates). A detailed morphological characterization (e.g., SEM or TEM) would be necessary to directly confirm the relationship between CNT dispersion/agglomeration and the observed hydration behavior.

### 4.2. EIS Results of Measurements in Liquid Conditions

**Figure 3:**
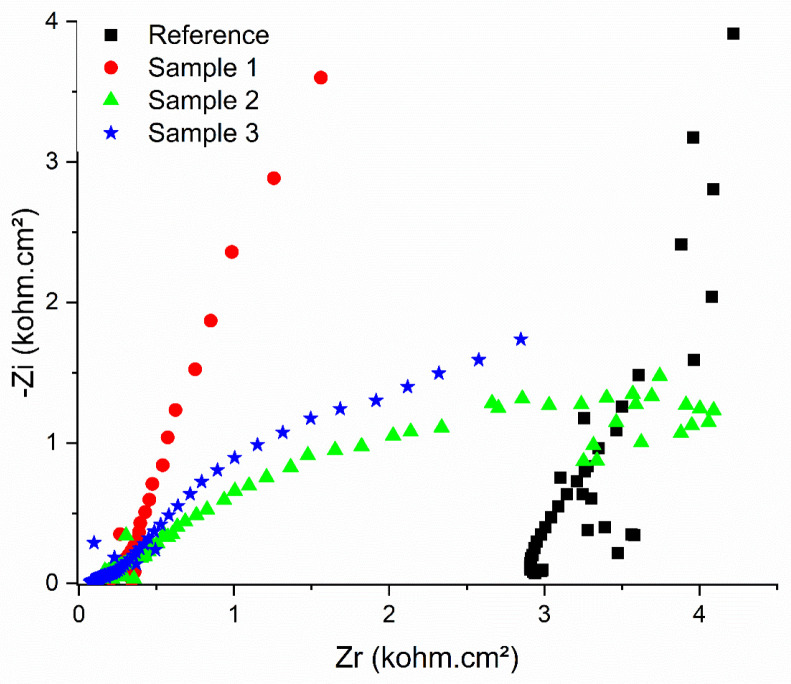
Raw EIS data as a Nyquist plot of the samples investigated in PBS.

**Figure 4:**
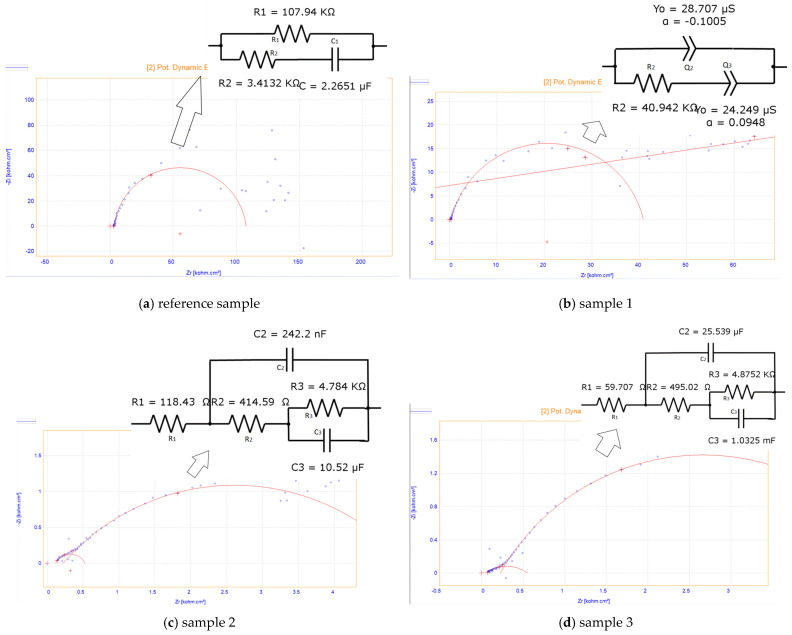
The equivalent circuits for the investigated samples.

The results of the measurements in liquid conditions are presented in Figure 3 as Nyquist plots, obtained in phosphate-buffered saline (PBS, pH 7.4), along with the corresponding fitting curves (solid red lines in Figure 3) generated using OrigaMaster5 software. All samples exhibit different behavior, yet they are consistent with the proposed equivalent electrical circuits (EECs), with χ^2^ values below 0.001 in each case. The reference sample, containing only the polymer, seems to exhibit inductive behavior at high frequencies (>10 kHz)—Figure 3; however, this behavior is a parasitic artifact produced by the experimental setup—Figure 4a. The EEC showed a semicircle at intermediate and low frequencies. A notable increase in electrical conductivity is observed due to the presence of CNTs, which reduces the total impedance by more than two orders of magnitude. In this way, the parasitic inductance is masked, and the dominant interfacial processes shift toward the resistive-capacitive regime.

The behavior of the samples with different CNT loadings is as follows:

(i) Sample 1 exhibits a single semicircle at high and intermediate frequencies, followed by linear behavior in the low-frequency range—Figure 4b.
(ii) Sample 2 exhibits two semicircles at high and medium-low frequencies—Figure 4c.
(iii) Sample 3 exhibits two semicircles (Figure 4d). The different impedance behavior is related to the different CNT loading of the polymer; more specifically, the porosity of the composite significantly influences the spectra. The reasoning is that, by adding CNTs to the polymer, we increased its conductivity and reduced its resistance.

In the Nyquist plots, the X_min_ indicates the electrolytic resistance, which is the resistance of the electrolytic solution and the solution in the pores of the hydrogel. Considering that all the samples were conditioned in identical solutions (PBS, pH = 7.4), which insured the same ionic concentration, type of ions, and temperature and were measured in the same configuration and have similar areas (not to mention, the electrolyte solution and the conditioning solution are the same), the variation in the X_min_ are attributed to the morphology and the doping with CNT of the hydrogel.

Thus, Table 3 indicates the variation in the X_min_ with the samples. The main observation is that the higher the concentration of CNT loading, the lower X_min_ is: 3.309 (reference sample, 0% CNT loading) > 0.3079 (Sample 1, 1% CNT loading) > 0.1184 (Sample 2, 2% CNT loading) > 0.059 (Sample 3, 3% CNT loading) [32]. Samples 2 and 3 have the most complex impedance among the samples: two semicircles can be observed with an equivalent circuit consisting of 3 resistors and 2 capacitors corresponding to the capacitive and resistive properties of the hydrogel (double-layer capacitance and Rct), as well as surface characteristics. In correlation with the hydration capacity, we observe that for Sample 3, the experimental data show the highest hydration and the lowest resistance. This can be explained by the compactness and structural uniformity of the composite [11]. Table 3 summarizes the impedance parameters, and the equivalent circuits are presented in Figure 4.

Table 3 summarizes the impedance parameters, and the equivalent circuits are presented in Figure 4.

In Table 3, the diameter of the semicircle is attributed to the charge-transfer resistance (R_p_), which accounts for the resistance of the supporting electrolyte, the resistance of the composite materials (polymer matrix + electrolyte solution in the pores of the polymer), and the contact resistance between the composite material and current collector (alligator clips, in our case).

For all our samples, the value of R_P_ is higher than that of the R_s_. However, the value for the samples containing CNT is lower than that for the reference polymer, which indicates that the incorporation of CNTs in the hydrogels offers a better interfacial contact and faster ion transport [32].

The formation of a conductive network is supported by the inverse proportionality between the progressive decrease in the values of Rs and Rct, which leads to an increase in the CNT content. Sample 3 (3% by weight CNT) is characterized by the lowest total resistance (~0.84 Ω·cm^2^) and the highest CPE value, which indicates and supports faster ion transport and superior interfacial capacitance. The CPE is a constant-phase element representing the non-ideal capacitance of the double layer (*n* ≈ 0.8–0.95 indicates moderate surface heterogeneity) and, in Table 3, is represented as a correlation.

The results presented are fully consistent with the data regarding hydration capacity. Sample 3 exhibits the highest degree of water absorption, which facilitates ion mobility. At the same time, well-dispersed CNTs ensure efficient electronic pathways, resulting in the lowest charge-transfer resistance and capacitive behavior closest to the ideal (*n* closest to one).

### 4.3. EIS Results of Measurements Taken Under Dry Conditions

**Figure 5:**
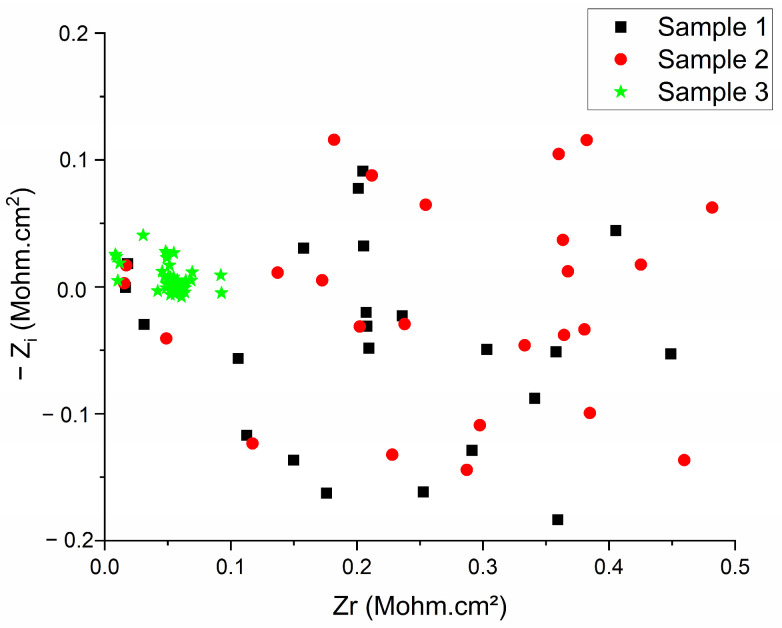
EIS of the samples under dry conditions.

The impedance measured by EIS under dry conditions (without external electrolyte) supports, from one perspective, the EIS results obtained in the liquid medium (Figure 4), as it shows that Sample 3 has the highest conductivity compared to the other samples. However, a radically different behavior is observed compared to the measurements performed in a liquid medium (PBS), as the absence of an electrolyte affects the hydrogel’s performance. This behavior is supported by Figure 5, where the complete EIS spectrum cannot be fitted to any known equivalent circuit, and the impedance value of all samples increased by several orders of magnitude, reaching the MΩ·cm^2^ range. Their observable distribution within the Nyquist plots is random, with highly dispersed, non-reproducible data points that could not be satisfactorily fitted to any conventional equivalent electrical circuit (χ^2^ values > 0.1), indicating a highly porous structure.

This “randomness” of the EIS data in dry conditions was supported by the fitting of the weighted residuals, which are randomly scattered around the zero axis with no discernible trend. The main source for the random distribution is the low signal-to-noise ratio (SNR), which occurs due to a lack of electrical conductivity, which, in turn, translates to a very large impedance (approaching the instrument’s limit). This lack of fit between the equivalent circuit model and measured data is produced by the presence of large and/or numerous defects and pores [33]. Figure 5 shows that random defects and pores strongly contribute to EIS data variability, disrupting the electrical conductivity of the polymeric composite matrix and increasing resistance for Samples 1 and 2, in the MΩ·cm^2^ range. However, this porous structure is connected and conductive for Sample 3 (as seen in Figure 5), indicating that the signal is conducted by the CNT–polymer cross-linked chains.

The impedance measured by EIS under dry conditions (without external electrolyte) shows that Sample 3 maintains the lowest resistance compared to the other samples. However, a radically different behavior is observed compared to the liquid medium (PBS). The impedance of all samples increased by several orders of magnitude, reaching the MΩ·cm^2^ range. As shown in Figure 5, the data points are highly dispersed and random, and could not be satisfactorily fitted to any conventional equivalent circuit model (χ^2^ > 0.1). This variability is likely caused by imperfect electrode–hydrogel contact and the complete absence of electrolyte, resulting in highly unstable interfaces.

We acknowledge that such non-reproducible behavior under dry conditions precludes the current formulation from being considered a reliable sensor for medical or high-fidelity EEG monitoring at this stage. Although the 3 wt% SWCNT sample showed the lowest relative resistance, suggesting the presence of a percolating conductive network, these results only indicate the potential robustness of the CNT network. They do not demonstrate the material’s ability to reliably capture microvolt EEG signals.

To properly evaluate the “high-fidelity” potential of the material, future work will include benchtop tests using simulated microvolt EEG signals (e.g., sinusoidal signals at 1–40 Hz with amplitudes of 5–50 µV) as well as real EEG recordings (alpha rhythm attenuation and eye-blink artifacts) with a prototype electrode integrated into an OpenBCI system. These experiments will allow quantitative assessment of signal-to-noise ratio (SNR) and direct comparison with conventional Ag/AgCl electrodes.

### 4.4. Implications for Real Intra-Aural EEG Applications

The ear canal is a semi-dry microenvironment where earwax (cerumen) is present, resulting in low and fluctuating humidity. Therefore, the EIS measurements under dry conditions represent the most unfavorable scenario that must be considered for prolonged wear, when partial dehydration of the hydrogel may occur. In dry conditions, the impedance of all samples increased by several orders of magnitude, reaching the MΩ·cm^2^ range. The data points were highly dispersed and could not be satisfactorily fitted to any conventional equivalent circuit model (χ^2^ > 0.1). This behavior highlights the strong sensitivity of the hydrogel to dehydration and represents the most unfavorable scenario for intra-aural EEG applications. Given that typical EEG acquisition requires electrode-skin impedances below 5–10 kΩ, the current formulation cannot yet be considered functional as a standalone solid electrode in fully dry conditions.

Nevertheless, even in the absence of an external electrolyte, the 3 wt% SWCNT sample maintained the lowest relative resistance among the tested compositions, suggesting the presence of a percolating CNT-based conductive network. While this indicates potential robustness of the conductive network, it does not demonstrate the material’s ability to reliably capture microvolt EEG signals. Maintaining a minimal level of hydration remains critical for practical performance. Consequently, the incorporation of humectants (such as glycerol or binary water-glycerol mixtures) is not only a future optimization but a necessary component for achieving stable, long-term operation in the ear canal microenvironment.

Although PBS provides a useful baseline for electrochemical characterization, it does not account for the presence of cerumen, a natural insulator that can dramatically increase electrode-skin impedance. Future studies will incorporate more realistic testing interfaces, including synthetic sebum and cerumen-coated surfaces, to better evaluate the performance of the hydrogel electrodes under the inherent challenges of the intra-aural environment.

## 5. Discussion

Electrochemical impedance spectroscopy (EIS) is a powerful tool for modeling the electrochemical processes occurring at the electrode–electrolyte interface through equivalent circuit models. However, due to the complexity of the SWCNT-doped GelMA/HEMA composite, a single equivalent circuit cannot fully describe all observed features.

In the high-frequency range, the undoped hydrogel (Sample 1) exhibits an inductive component, which disappears upon incorporation of SWCNTs and is replaced by a semicircle. In the intermediate frequency range, all samples show semicircular behavior. From the diameter of the semicircle, the resistance of the electrolyte solution inside the pores can be extracted. This resistance decreases significantly with increasing SWCNT content—from several kΩ in the undoped and low-concentration samples to approximately 0.72 Ω·cm^2^ in Sample 3. This indicates that the presence of SWCNTs markedly enhances the ionic conductivity within the hydrogel matrix.

The charge-transfer resistance (Rct) at the hydrogel/electrolyte interface also decreases progressively with SWCNT loading, reaching the lowest value for Sample 3. Since Rct is lower than the solution resistance, the 3 wt% SWCNT hydrogel demonstrates superior interfacial conductance and faster ion transport compared to the other formulations.

These electrochemical improvements correlate well with the hydration capacity results: Sample 3 exhibits both the highest hydration and the lowest overall impedance. This behavior is consistent with the formation of a more effective conductive percolating network at 3 wt% SWCNT loading.

In dry conditions, the impedance of all samples increased by several orders of magnitude, reaching the MΩ·cm^2^ range. The data points became highly dispersed and could not be satisfactorily fitted to conventional equivalent circuits (χ^2^ > 0.1). This drastic increase highlights the strong sensitivity of the hydrogel to dehydration and represents the most unfavorable scenario for intra-aural applications. Given that typical EEG acquisition requires electrode-skin impedances below 5–10 kΩ, the current formulation cannot yet be considered functional as a standalone solid electrode in fully dry conditions. Nevertheless, Sample 3 maintained the lowest relative resistance among the tested compositions, suggesting the presence of a percolating CNT-based conductive network even in the absence of an external electrolyte.

## 6. Conclusions and Future Research Directions

This study examined GelMA/HEMA/SWCNT composite hydrogels from an electrochemical perspective, with the aim of developing advanced materials for intra-aural EEG electrodes. Building upon preliminary cytocompatibility and mechanical characterization reported in our previous work [26], the present study provides a systematic evaluation of hydration capacity and a detailed electrochemical impedance spectroscopy (EIS) analysis under both liquid (PBS) and dry conditions.

The results demonstrate that the 3 wt% SWCNT formulation exhibits the lowest charge-transfer resistance, the highest double-layer capacitance, and the highest hydration capacity among the tested compositions. These properties indicate the formation of a more effective conductive percolating network compared to lower SWCNT loadings and the undoped hydrogel, making the 3 wt% SWCNT hydrogel the most promising composition identified in this study. Although the 3 wt% SWCNT hydrogel exhibits promising electrochemical properties (i.e., low charge-transfer resistance and high hydration capacity), its ability to deliver high-fidelity EEG signals remains to be demonstrated. The material cannot yet be considered validated for EEG applications, as neither real nor simulated EEG signal acquisition has been performed.

Future research will focus on developing these hydrogels via 3D printing of customized in-ear electrodes and integrating them into a functional in-ear EEG prototype. The performance of the resulting system will be validated through comparative testing against conventional Ag/AgCl electrodes using an open-source OpenBCI EEG system, under both controlled laboratory conditions and real-world settings. Key evaluation parameters will include the ability to capture basic neural signals (e.g., alpha rhythm attenuation and eye-blink artifacts), signal-to-noise ratio (SNR), temporal correlation with scalp EEG recordings, resistance to motion artifacts, and long-term hydration stability (potentially enhanced through the incorporation of humectants). Additional validation will assess the insulating effects of cerumen using synthetic sebum and cerumen-coated interfaces.

## Figures and Tables

**Table 1:** Composition of the GelMA/HEMA hydrogel formulations doped with single-walled carbon nanotubes. Adapted from [26].

Sample ID	Base Composition	CNT Concentration
Control	GelMA + HEMA	0% (no CNT)
Sample 1		1% CNT
Sample 2		2% CNT
Sample 3		3% CNT

**Table 2:** Weight of samples before and after conditioning in phosphate-buffered saline (PBS).

Samples	Initial Mass (g)	Mass of Samples After Hydration (g)	Degree of Hydration (%)	Hydration Capacity (%/cm^2^)
Reference	0.24215	0.34155	29.1	7.2
Sample 1	0.21673	0.30278	28.4	7.1
Sample 2	0.35293	0.45182	21.8	5.4
Sample 3	0.21006	0.29915	29.7	7.4

The hydration capacity was calculated for 1 cm^2^ because the original samples had different dimensions; in order to average out the surface of the samples, we used a roughness factor of 2.1 to multiply the geometrical area of the samples and estimate the real surface of the samples.

**Table 3:** Circular regression parameters extracted from EIS measurements for the samples investigated in the intermediate region.

EIS Parameters (kΩ × cm^2^)	Reference	Sample 1	Sample 2	Sample 3
Center X	55.63	20.63	0.3257	0.30
Y-center	−6.315	−4.748	−0.1031	−0.34
Diameter	105.4	41.73	0.4631	0.84
Correlation	0.987	0.97	0.999	0.99
Depletion angle	−3.44°	−6.53°	−12.9°	−23.8°
X_min_	3.309	0.3079	0.1184	0.059

## Data Availability

The research data used in this article are available from the first author upon request.
